# Efficacy and safety of electrical cardioversion and catheter ablation during pregnancy: a case review and literature analysis

**DOI:** 10.3389/fcvm.2026.1786261

**Published:** 2026-06-24

**Authors:** Wentao Li, Jianyu Hu, Haiqi Song, Xiang Fang, Jiaming Liu, Liang Zhang, Hao Wang, Ruhui Liu, Rui Zeng

**Affiliations:** 1 Department of General Internal Medicine, West China Second University Hospital, Sichuan University, Chengdu, China; 2 Key Laboratory of Birth Defects and Related Diseases of Women and Children (Sichuan University), Ministry of Education, Chengdu, China; 3 Department of Cardiology, West China Hospital, Sichuan University, Chengdu, China

**Keywords:** catheter ablation, electrical cardioversion, pregnancy, paroxysmal supraventricular tachycardia, atrial fibrillation

## Abstract

**Background:**

Despite guideline recommendations, electrical cardioversion and catheter ablation remain underutilized for the management of pregnancy-associated arrhythmias, primarily due to persistent but unfounded safety concerns. However, delayed rhythm control may precipitate hemodynamic compromise, increasing risks of maternal cardiovascular instability and fetal distress. Herein, we report an illustrative case from our institution and provide a structured narrative review of electrical cardioversion and catheter ablation during pregnancy.

**Methods:**

We present a case of recurrent arrhythmia in pregnancy that was refractory to standard therapies. The patient failed to respond to both radiofrequency ablation and electrical cardioversion. A search of Web of Science, Embase, and PubMed identified relevant studies using the terms pregnancy, electrical cardioversion, and catheter ablation. The quality of included studies was assessed using the Joanna Briggs Institute (JBI) appraisal tools. The primary efficacy endpoint was successful restoration of sinus rhythm, while the safety endpoint focused on maternal-fetal adverse events.

**Results:**

A systematic review identified 19 studies (58 patients) reporting electrical cardioversion and 44 studies (159 patients) reporting catheter ablation during pregnancy, with success rates of 89.7% and 97.5%, respectively. Among pregnant women undergoing electrical cardioversion, there were 5 cases of preterm delivery and 2 cases of maternal-fetal mortality. The catheter ablation group had 2 cases of preterm delivery with no maternal-fetal mortality. Among different arrhythmia types, atrial fibrillation was the most common in the cardioversion group, while AVNRT predominated in the catheter ablation cohort.

**Conclusion:**

Both electrical cardioversion and catheter ablation are safe and effective treatment options during pregnancy. The available clinical evidence, though largely from case reports, consistently supports their use in this patient population.

## Introduction

Arrhythmias affect 0.5–1.2 per 1,000 pregnancies (1, 2). While most cases are benign, delayed intervention can lead to severe complications (3). Notably, arrhythmias contribute to 10.7% of maternal cardiovascular deaths (4). Pharmacological options are limited by Category C drug risks, while electrical cardioversion or catheter ablation lack robust safety data (5).

Current guidelines suggest that electrical cardioversion or catheter ablation may be considered during pregnancy (6). However, evidence on their safety and efficacy remains limited to isolated case reports or small series (7). There is a critical lack of comprehensive data to determine which specific arrhythmia cases in pregnant women warrant these procedures. Additionally, systematic evaluation of their effectiveness and safety profiles is lacking. This evidence gap contributes to clinical uncertainty among obstetricians and cardiologists managing pregnant patients with arrhythmias.

Although these interventions may pose risks such as fetal arrhythmia, preterm labor, or distress, timely cardioversion in emergencies can reduce complications (8). To address these knowledge gaps, a systematic review of electrical cardioversion and catheter ablation during pregnancy is urgently needed.

## Case

A 24-year-old primigravida presented with supraventricular tachycardia (SVT) on electrocardiogram during her 7-week prenatal examination. Her examination indicated normal thyroid function and cardiac function. Given the absence of clinical symptoms (no palpitations, chest discomfort), a conservative monitoring approach was adopted. At 28 weeks of gestation, the patient began experiencing exertional palpitations and fatigue. 24-hour Holter monitoring revealed SVT. Given concerns regarding antiarrhythmic drug-related adverse effects, the patient elected to proceed with catheter ablation therapy. Three-dimensional electroanatomic mapping (CARTO, Biosense Webster, Inc., Diamond Bar, CA) localized the earliest activation site of atrial tachycardia to the basal aspect of the right atrial appendage. Despite procedural challenges, the patient successfully achieved sinus rhythm with significant symptomatic improvement following zero-fluoroscopy radiofrequency ablation. At 35 weeks of gestation, three months following the ablation procedure, the patient presented with recurrent palpitations, and an electrocardiogram demonstrated PSVT. Intravenous adenosine triphosphate administration failed to achieve cardioversion, prompting cesarean delivery at 36 weeks after fetal lung maturation. The patient delivered a normal preterm infant. Postpartum electrocardiogram showed recurrent atrial tachycardia. Electrical cardioversion at 200J was also unsuccessful. The patient was subsequently placed on oral metoprolol and amiodarone therapy, and at her 3-month postpartum follow-up, the electrocardiogram indicated sinus rhythm.

## Methods

To ensure transparency and reproducibility, this systematic review was prospectively registered in PROSPERO (ID: CRD420251140058). We conducted comprehensive searches of PubMed, Embase, and Web of Science databases using Medical Subject Headings (MeSH) terms and keywords including “pregnancy”, “electrical cardioversion”, and “catheter ablation”. The complete search strategy is available in Supplemental Material.

We included studies reporting electrical cardioversion or catheter ablation performed during pregnancy. Studies involving pharmacological treatment alone or procedures conducted outside pregnancy were excluded. The primary endpoints were successful cardioversion (defined as restoration of sinus rhythm on the final attempt, irrespective of prior unsuccessful attempts) and maternal/fetal adverse outcomes. Additional methodological details are provided in Supplemental Material.

Study quality was rigorously assessed using appropriate tools: retrospective studies were evaluated with the Joanna Briggs Institute (JBI) checklist for cohort studies, while case reports were assessed using the JBI case report checklist. Complete quality assessment results are provided in Supplemental Material, with representative examples in the main text. No studies were excluded for low quality. Data extraction encompassed study characteristics, maternal demographics, gestational age at intervention, arrhythmia details, and maternal-fetal outcomes. Our primary endpoints were procedural success (defined as sinus rhythm restoration on final cardioversion attempt) and occurrence of severe adverse events. To maintain methodological rigor, all processes were performed independently by two investigators with cross-validation. Any discrepancies were resolved through consensus discussion to ensure accuracy and consistency in our review process.

To analyse the extracted data, we used SPSS version 25.0 (IBM Corp, Armonk, NY, USA). The measurement data were expressed as mean and were compared by independent t test, while the categorical data were expressed as numbers and percentages. And the categorical data were compared by *Χ*^2^ test with continuity correction. When comparing intergroup differences between the successful cardioversion group and the failed cardioversion group, continuous variables were analyzed using Welch's corrected t-test, while categorical variables were assessed with Fisher's exact test. *P* < 0.05 was considered of statistical significance.

## Results of the search

### Summary of included studies

**Figure 1:**
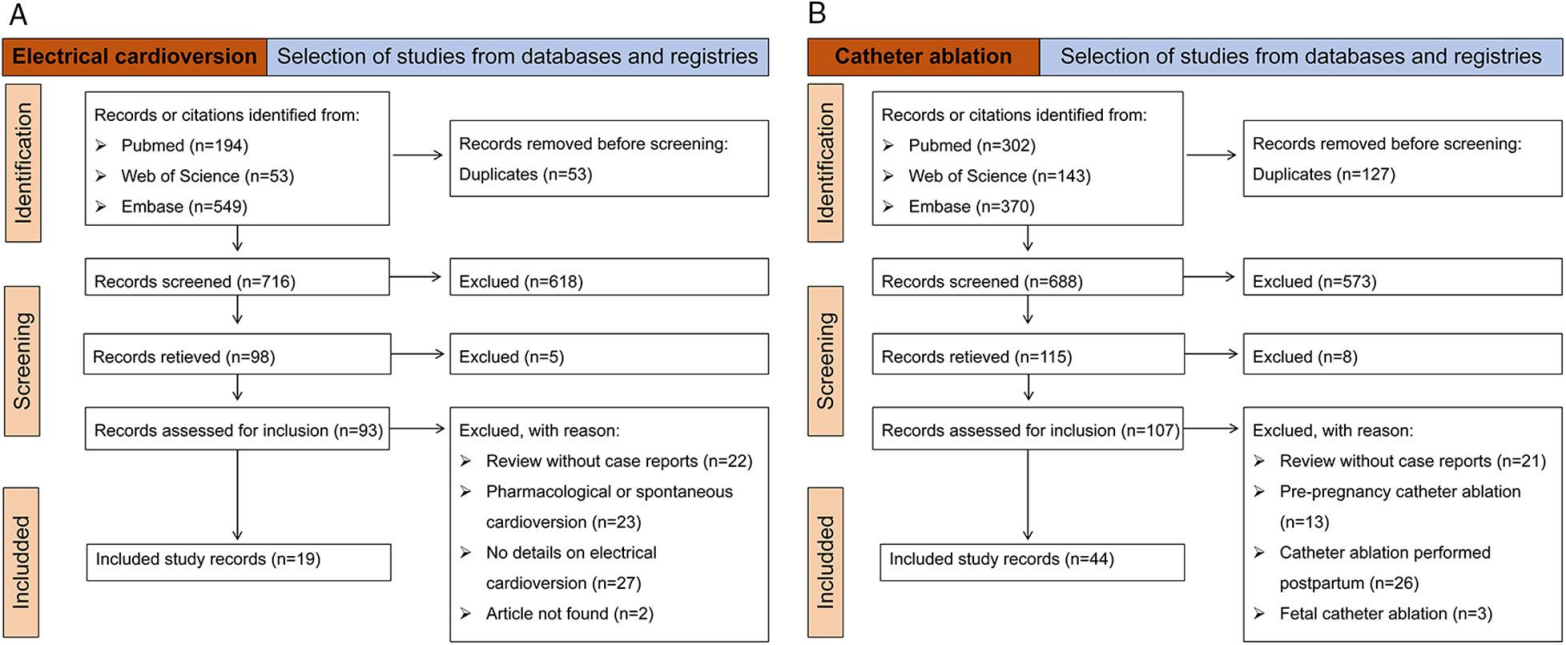
Study selection flow diagram.

Our systematic search identified 716 potentially eligible studies on electrical cardioversion during pregnancy, of which 19 studies (16 case reports, 2 case series, and 1 retrospective study) met the inclusion criteria after rigorous screening (Figure 1A). For catheter ablation, 44 (32 case reports, 8 case series, and 4 retrospective studies) out of 688 identified studies were included, encompassing a total of 159 procedures (Figure 1B).

### Electrical cardioversion group

The electrical cardioversion cohort comprised 58 pregnant women with the following clinical characteristics: mean maternal age was 30.3 ± 4.1 years, mean gestational age was 24.9 ± 8.3 weeks, and 52.2% were primiparous. Cardiac arrhythmias requiring cardioversion included atrial fibrillation (AF, 46.5%; 27/58), atrial flutter (AFL, 24.1%; 14/58), and supraventricular tachycardia (SVT, 15.5%; 9/58). The delivered energy ranged from 20-360 J (most commonly 100 J), with 82.8% of patients receiving pre-procedural pharmacotherapy. Comorbidities were prevalent, including congenital heart disease (36.2%, 21/58) and pre-existing arrhythmia history (31.0%, 18/58) (Table 1). A systematic summary of key variables from post-2000 case reports on pregnancy cardioversion is presented in Supplementary Table S1.

**Table 1:** Characteristics of pregnant women undergoing electrical cardioversion.

Variable	*N* = 58
Parity 0	24/46 (52.2)
Parity 1	19/46 (41.3)
Parity ≥2	3/46 (6.5)
Type of arrhythmia	
SVT	9 (15.5)
AT	5 (8.6)
AF	27 (46.5)
AFL	14 (24.1)
VT	2 (3.4)
VF	1 (1.7)
Electrical cardioversion energy	
SVT	20–200J
AT	100–360J
AF	50–250J
AFL	40–100J
VT	100–360J
VF	300J
Maternal age (y)	30.3
Gestation at cardioversion (week)	24.9
Gestation at birth (week)	36.6
Medical treatment initially	48 (82.8)
Unstable haemodynamics	7 (12.1)
History of arrhythmia	18 (31.0)
Congenital heart disease	21 (36.2)

SVT, supraventricular tachycardia; AT, atrial tachycardia; AF, atrial fibrillation; AFL, atrial flutter; VT, ventricular tachycardia; VF, ventricular fibrillation.

### Efficacy and safety outcomes of electrical cardioversion

Electrical cardioversion demonstrated an overall success rate of 89.7% among enrolled pregnant patients. Of the six unsuccessful cases, three involved atrial tachycardia (AT), one ventricular tachycardia (VT), one AF, and one SVT. Adverse outcomes included preterm delivery (8.6%, 5/58), maternal-fetal mortality (3.4%, 2/58), and small-for-gestational-age infant (1.7%, 1/58) (Table 2).

**Table 2:** Efficacy and safety of electrical cardioversion.

Variable	*N* = 58
Successful cardioversion	52 (89.7)
Recurrence	6 (10.3)
SGA	1 (1.7)
Congenital malformations	0
Preterm birth	5 (8.6)
Arrhythmia complications	0
Maternal and fetal death	2 (3.4)

SGA, small for gestational age.

### Comparison of outcomes between failed and successful electrical cardioversion

Patients in the failed cardioversion group were significantly younger than those in the successful group (*P* = 0.021). No significant between-group differences were observed in gestational age at cardioversion, history of cardiac disease, primiparity, or use of antiarrhythmic medications (all *P* > 0.05) (Table 3).

**Table 3:** Comparison between the electrical cardioversion failure group and the success group.

Variable	Failure (*n* = 6)	Success (*n* = 52)	*P*
Gestation at cardioversion	25.5 ± 9.9	26.5 ± 5.1	0.682
Age	25.3 ± 3.7	30.2 ± 3.1	**0**.**021**
Parity 0	5 (83.3)	19 (36.5)	0.101
Atrial fibrillation	1 (16.7)	27 (51.9)	0.102
Antiarrhythmic drug	5 (83.3)	24 (46.2)	0.085
Previous history of cardiac disease	1 (16.7)	21 (40.4)	0.267

Patients in the failed cardioversion group were significantly younger than those in the successful group (*P* < 0.05).

### Catheter ablation group

The majority of pregnancies (72.3%, 115/159) underwent the intervention during the second or third trimester. The cohort included atrioventricular nodal reentrant tachycardia (AVNRT; 23.9%, 38/159), premature ventricular contractions (PVC; 20.1%, 32/159), and atrial tachycardia (AT; 16.4%, 26/159). Three-dimensional electroanatomic mapping systems were employed in all cases: CARTO (55.3%, 88/159) and EnSite NavX (29.6%, 47/159). Radiofrequency ablation was the primary modality (98.7%, 157/159), with cryoablation used in two selected cases (1.3%). Fluoroscopy was avoided in 86.8% of procedures (138/159), with low-dose fluoroscopic guidance required in the remaining 13.2% (21/159) (Table 4). Supplementary Table S2 compiles key procedural variables from some cases of pregnancy catheter ablation.

**Table 4:** Characteristic of pregnant women undergoing catheter ablation.

Variable	*N* = 159
Maternal age (y)	29.8
Gestation at ablation (week)	22.7
Trimester	*N* = 65
First trimester	9 (13.8)
Second trimester	36 (55.4)
Third trimester	20 (30.8)
Arrhythmia type	
AVNRT	38 (23.9)
AVRT	7 (4.4)
AT	26 (16.4)
PVC	32 (20.1)
VT	16 (10.1)
WPW	13 (8.2)
AF/AFL	9 (5.7)
Others	18 (11.3)
Mapping system	*N* = 147
CARTO	88 (59.9)
EnSite NavX	47 (32.0)
Others	12 (8.1)
Zero-fluoroscopy	138 (86.8)
Under-fluoroscopy	21 (13.2)
Radiofrequency cather ablation	157 (98.7)
Cyoablation	2 (1.3)
History of arrhythmia	26 (16.4)

AVNRT, atrioventricular nodal reentrant tachycardia; SVT, supraventricular tachycardia; AT, atrial tachycardia; AF, atrial fibrillation; AFL, atrial flutter; VT, ventricular tachycardia; PVC, premature ventricular contraction; WPW, Wolff Parkinson White; SGA, small for gestational age.

### Efficacy and safety outcomes of catheter ablation

Catheter ablation demonstrated a 97.5% (155/159) success rate in pregnant patients, with arrhythmia recurrence occurring in 4 cases (2.5%): AT (*n* = 2), SVT (*n* = 1), and Wolff-Parkinson-White syndrome (*n* = 1). Adverse outcomes included preterm delivery (1.2%, 2/159) and small-for-gestational-age infant (0.6%, 1/159), with no maternal or fetal mortality (Table 5). Comparative analysis between failed and successful ablation procedures was precluded by the significant sample size imbalance (4 recurrence cases vs. 155 non-recurrence cases).

**Table 5:** Procedural efficacy and safety of catheter ablation.

Variable	*N* = 159
Successful cardioversion	155 (97.5)
Recurrence	4 (2.5)
SGA	1 (0.6)
Congenital malformations	0
Preterm birth	2 (1.2)
Arrhythmia complications	0
Maternal and fetal death	0

## Discussion

The unique electrophysiological changes during pregnancy - including increased cardiac output, hormonal fluctuations, and altered drug metabolism - collectively increase both arrhythmia susceptibility and therapeutic resistance (7). Given the elevated risk of maternal-fetal complications, prompt arrhythmia management is essential in this population (9). Our analysis of published case reports indicates that both electrical cardioversion and catheter ablation are effective and safe during pregnancy. These two approaches represent distinct therapeutic strategies, each appropriate for specific clinical situations. For pregnant patients with hemodynamic instability, electrical cardioversion is the treatment of choice irrespective of arrhythmia type and may also serve as adjunctive therapy when pharmacological management fails. For hemodynamically stable patients, catheter ablation demonstrates superior efficacy, particularly for managing supraventricular tachycardia and premature ventricular contractions during pregnancy.

Electrical cardioversion during pregnancy is indicated for hemodynamically unstable arrhythmias or drug-refractory episodes (10). Although the incidence of pregnancy-associated arrhythmias increases with gestational age, cardioversion demonstrates consistent safety across all trimesters. The reported case of severe fetal and maternal mortality derived from historical case reports and were attributed to delayed intervention, which was initiated only after overt clinical deterioration accompanied by hemodynamic instability (11). A critical consideration in this population is the mandatory anteroposterior electrode placement to minimize fetal current exposure (12). Given the higher fibrillation threshold of fetal myocardium and the minimal current exposure, the procedure carries negligible fetal risk (13). Due to procedural discomfort, deep sedation or general anesthesia is typically required (14). In case of acute hemodynamic instability, an initial energy of 50–100 J is recommended, with gradual escalation up to 400 J if necessary.

Catheter ablation represents a potentially curative approach for pregnancy-related tachyarrhythmias, particularly in cases refractory to pharmacotherapy or with high recurrence rates despite electrical cardioversion (15). While zero-fluoroscopy protocols remain mandatory, minimized radiation exposure with shielding may be employed when absolutely necessary (16). Although technically feasible during all gestational stages, the second and third trimesters are preferred due to demonstrated maternal-fetal safety. Optimal outcomes require a specialized multidisciplinary team, including electrophysiologists, obstetricians, and perinatal anesthesiologists (17).

Our findings demonstrate that electrical cardioversion is more frequently utilized for AF, whereas catheter ablation is predominantly indicated for AVNRT. In the management of pregnancy-associated arrhythmias, neither electrical cardioversion nor catheter ablation should constitute first-line therapy. The optimal approach requires comprehensive evaluation of gestational age, hemodynamic status, and arrhythmia type. SVT is the most common pregnancy-related arrhythmia, with vagal maneuvers representing first-line therapy and adenosine serving as the preferred pharmacologic agent owing to its rapid onset and short half-life (12). Non-dihydropyridine calcium channel blockers show comparable efficacy to adenosine while providing superior safety profiles, rendering them a viable alternative (18). For hemodynamically unstable patients, electrical cardioversion demonstrates high efficacy and should be expeditiously implemented (19). In refractory or recurrent cases, catheter ablation emerges as a viable intervention and represents an effective approach for preventing recurrent supraventricular tachycardia in pregnant women.

AF during pregnancy most frequently occurs in association with structural heart disease, hyperthyroidism, or advanced maternal age (20). Initial management focuses on rate control, with beta-blockers representing first-line therapy. Electrical cardioversion constitutes a safe option for acute rhythm control; notably, if AF persists beyond 48 h, transesophageal echocardiography must be performed to exclude left atrial thrombus prior to cardioversion (21). Anticoagulation in pregnancy presents unique challenges. Low-molecular-weight heparin is preferred given contraindications to oral anticoagulants, although the overall thromboembolic risk in this population remains low (20). Catheter ablation may be considered for patients with recurrent symptomatic arrhythmias or based on patient preference. This intervention demands multidisciplinary coordination to minimize maternal and fetal risks, encompassing preprocedural optimization- including protocol-guided anticoagulation for AF and real-time fetal monitoring (22).

Among pregnant women presenting with ventricular arrhythmias, PVC occur significantly more frequently than ventricular tachycardia (VT). Evidence suggests that in structurally normal hearts, PVC burden shows no significant association with adverse maternal or neonatal outcomes (23). Moreover, antiarrhythmic therapy demonstrated no superior clinical outcomes compared to no treatment. A substantial proportion of pregnancy-associated VT cases are benign and often spontaneously convert to sinus rhythm after delivery (24). Therapeutic options should be deferred until the second trimester: *β*-blockers for PVC management, and lidocaine/verapamil for VT (9, 25). Electrical cardioversion effectively terminates VT; however, its success rate is substantially lower than for SVT, often necessitating repeated attempts. Catheter ablation represents another viable treatment option for both VT and PVC, with success rates related to the anatomical origin of the arrhythmia (26, 27).

Pregnant women with structural heart disease face increased risks of malignant arrhythmias. Due to radiation exposure concerns, implantable cardioverter-defibrillators (ICD) implantation during pregnancy is exceptionally rare, though documented in case reports (28). Compared with ICD, wearable cardioverter-defibrillators (WCD) provide superior safety profiles while enabling uneventful pregnancy in women at risk for sudden cardiac death through interdisciplinary monitoring and pharmacotherapy (29).

In pregnant patients, electrical cardioversion demonstrates comparable success rates to those in non-pregnant populations. Evidence indicates a significantly higher incidence of fetal adverse events in the fluoroscopy group compared to zero-fluoroscopy procedures. Notably, subgroup analysis revealed a 14.3% adverse event rate among cases with radiation exposure >50 mGy, which accounted for one-third of exposed patients (30, 31). These findings strongly support the use of zero-fluoroscopy techniques to avoid fetal radiation risks. Although data on zero-fluoroscopy ablation during pregnancy remain limited, advances in 3D mapping and intracardiac echocardiography have enabled consistent radiation-free procedures for this population (32). Moreover, the use of a visualizable steerable sheath significantly reduces fluoroscopy exposure without prolonging procedure time, thereby decreasing risks for both mother and fetus during catheter ablation (33–35). This approach facilitates arrhythmia management in early gestation, providing a safer alternative to pharmacotherapy given first-trimester risks. Beyond mapping techniques, novel ablation modalities including pulsed-field ablation have achieved substantial clinical adoption. Nevertheless, further research is warranted to assess the safety and efficacy of these emerging technologies in pregnant populations.

### Limitation

The low incidence of arrhythmias necessitating electrical cardioversion or catheter ablation during pregnancy inherently limited the sample size. While this accurately mirrors real-world clinical practice, the interpretation of findings warrants caution due to potential selection bias and the lack of comparator groups. The existing evidence consists of case reports spanning several decades, with the majority published post-2000, and these methodological variations in reporting appear to have minimal impact on primary outcome assessments.
