# Supplementary material for: Efficacy and safety of electrical cardioversion and catheter ablation during pregnancy: a case review and literature analysis
Source: Front Cardiovasc Med. 2026 Jun 24;13:1786261. doi: 10.3389/fcvm.2026.1786261 (PMC13359150; doi:10.3389/fcvm.2026.1786261)
Supplement: Supplementary file 1 [file Datasheet1.pdf]

Supplement to:

Efficacy and Safety of Electrical Cardioversion and Catheter Ablation During Pregnancy: A Case Review and Literature Analysis

Page 2-3: Search strategy of patients undergoing cardioversion during pregnancy

Page 4:Search strategy of patients undergoing catheter ablation during pregnancy

Page 5-6:Inclusion criteria, exclusion criteria and data extraction

Page 7-8: the Joanna Briggs Institute (JBI) checklist for cohort studies and JBI case report checklist

Page 9:Table S1: Characteristics of included studies reported in pregnancy women and electrical cardioversion was performed

Page 10: Table S2: Characteristics of included studies reported in women with arrhythmia and catheter ablation was performed

Page 11-13: References

PubMed

1. ((electrical cardioversion) AND (pregnancy)) AND (("1965/09/05"[Date - Publication] : "2025/09/05"[Date - Publication]))

Maternal and fetal outcomes

1. Pregnancy Outcome [Mesh]
2. Pregnancy Complications [Mesh]
3. Abortion, Induced [Mesh]
4. Obstetric Surgical Procedures [Mesh]
5. Delivery, Obstetric [Mesh]
6. Infant, Newborn [Mesh]
7. Infant, Low Birth Weight [Mesh]
8. Premature Birth [Mesh]
9. Congenital Abnormalities [Mesh]
10. Perinatal Mortality [Mesh]
11. Infant Mortality [Mesh]
12. Perinatal Death [Mesh]
13. Intensive Care, Neonatal [Mesh]
14. Infant, Newborn, Diseases [Mesh]
15. Infant, Premature [Mesh]
16. Fetal Diseases [Mesh]
17. Fetal Arrhythmia [Mesh]

Search results: 194 records

Web of Science

1. TS=(cardioversion)
2. TS=(electrical cardioversion)
3. TS=(preganancy)
4. DOP=(1965-09-05/2025-09-05)

Maternal and fetal outcomes

1. ALL=(pregnancy)
2. ALL=(gestational)
3. ALL=(pregnant)
4. ALL=(spontaneous abortion)
5. ALL=(induced abortion)
6. ALL=(congenital disorder)
7. ALL=(newborn)
8. ALL=(infant)
9. ALL=(premature delivery)
10. ALL=(fetal arrhythmia)

Search results: 53 records

Embase

('electrical cardioversion')/br AND ('pregnancy') AND [1965-2025]/py

Maternal and fetal outcomes

1. 'pregnancy':ab,ti
2. 'pregnancy'/exp
3. 'gestational':ab,ti
4. 'pregnant':ab,ti

Search results: 549 records

PubMed

2. ((catheter ablation) AND (pregnancy)) AND (("2000/01/01"[Date - Publication] : "2025/09/05"[Date - Publication]))

Maternal and fetal outcomes

1. Pregnancy Outcome [Mesh]
2. Pregnancy Complications [Mesh]
3. Abortion, Induced [Mesh]
4. Delivery, Obstetric [Mesh]
5. Infant, Low Birth Weight [Mesh]
6. Premature Birth [Mesh]
7. Congenital Abnormalities [Mesh]
8. Infant, Newborn, Diseases [Mesh]
9. Infant, Premature [Mesh]
10. Fetal Diseases [Mesh]
11. Fetal Arrhythmia [Mesh]

Search results: 302 records

Web of Science

1. TS=(ablation)
2. TS=(catheter ablation)
3. TS=(radiofrequency ablation)
4. TS=(cryoablation)
3. TS=(preganancy)
4. DOP=(2000-01-01/2025-09-05)

Maternal and fetal outcomes

1. ALL=(pregnancy)
2. ALL=(gestational)
3. ALL=(abortion)
4. ALL=(congenital disorder)
5. ALL=(infant)
6. ALL=(premature delivery)

Search results: 143 records

Embase

(' ablation')/br('catheter ablation')/br AND ('pregnancy') AND [2000-2025]/py

Maternal and fetal outcomes

1. 'pregnancy':ab,ti
2. 'pregnancy'/exp
1. 'fetus':ab,ti
2. 'fetus'/exp

Search results: 370 records

Inclusion criteria

We selected studies that were published; without language restrictions; and that met the following inclusion criteria (PICOS):

• Population: Pregnant women diagnosed with arrhythmia who underwent electrical cardioversion/catheter ablation.

• Intervention: electrical cardioversion/catheter ablation approved for the treatment of pregnant patients with arrhythmias during gestation.

• Comparison: not applicable.

• Outcomes: Whether conversion to sinus rhythm is achieved; pregnancy, foetal and neonatal outcomes.

• Study type: randomized controlled trials and non-randomized studies (cohort studies,case-control studies, case series, clinical case studies, and patient registries).

Exclusion criteria

• Reviews and animal studies.

• Pregnant women who underwent pharmacological cardioversion or spontaneous cardioversion, without receiving electrical cardioversion/catheter therapy.

• Electrical cardioversion or catheter ablation performed either prenatally or postpartum, outside of pregnancy.

• Exposure to pharmacological cardioversion alone.

• The original text cannot be retrieved.

• Repeated publications.

• The description solely mentions the administration of catheter ablation or electrical cardioversion, without providing any details or parameters regarding the treatment.

Data extraction

The following criteria during data extracted: (1) author(s) of the study; (2) publication year; (3) maternal age and obstetric history; (4) type of arrhythmia;(5) gestation at cardioversion and delivery;(6) symptoms; (7) whether antiarrhythmic drugs were used, the specific drug names and corresponding dosages; (8) whether hemodynamic abnormalities were present; (9) maternal comorbidities; (10) type of arrhythmia; (11) energy dose of electrical cardioversion; (12) catheter

ablation mapping system; (13) use of radiation; (14) energy selection for catheter ablation; (15) mode of delivery; (16) pregnancy, foetal and neonatal outcomes; (17) maternal comorbidities; For unclear details, the researchers will contact the corresponding authors by email for detailed information. The initial search was conducted by one investigator and validated by a secondary conductor to ensure accuracy of search and application of exclusion criteria

JBI checklist for cohort studies

Author and Year	Clear definition of study population	Representativeness of the sample	Objective measurement of exposure/intervention	Standardized outcome measurement	Sufficient follow-up duration for outcomes	Follow-up completeness	Statistical adjustment for confounders	Ethical approval and informed consent
2023M. Cauldwell	Y	Y	Y	Y	Y	Y	Y	Y
2019Meng-Meng Li	Y	Y	Y	Y	Y	Y	Y	Y
2017Edward Koźluk	Y	Y	Y	Y	Y	Y	Y	Y
2010Lukasz Szumowski	Y	Y	Y	N	Y	Y	Y	Y

Y,yes; N,no; U,unclear.

JBI case report checklist

Year and Author	Were patient's demographic characteristics clearly described?	Was the patient's history clearly described and presented as a timeline?	Was the current clinical condition of the patient on presentation clearly described?	Were diagnostic tests or assessment methods and the results clearly described?	Was the intervention or treatment procedure clearly described?	Was the post intervention clinical condition clearly described?	Were adverse events [harms] or unanticipated events identified and described?	Does the case report provide takeaway lessons?
2019 N. JanjuaB	Y	Y	Y	Y	Y	Y	Y	Y
2016 Matěcha, J	Y	N	Y	Y	Y	Y	Y	Y
2016 Rekha Agrawal	Y	Y	Y	Y	Y	Y	Y	Y
2016 Sungmin Lee	Y	Y	Y	Y	Y	Y	Y	Y
2014 N. Murphy	Y	Y	Y	Y	Y	Y	Y	Y
2012 Fevzi Yılmaz	Y	Y	Y	Y	Y	Y	Y	N
2011 A. DiCarlo-Meacham	Y	Y	Y	Y	Y	Y	Y	Y
2011 Charly J Sengheiser	Y	Y	Y	Y	Y	Y	Y	Y

2010 John D	Y	Y	Y	Y	Y	Y	Y	Y
2009 A Puri	Y	Y	Y	Y	Y	Y	Y	Y
2007 Chia-Hui Lin	Y	Y	Y	Y	Y	Y	Y	Y
2006 Arimie R	Y	N	Y	Y	Y	N	Y	N
2006 Tsui-Hua Wu	Y	Y	N	Y	Y	Y	Y	Y
2006 Parasuraman R	Y	Y	Y	Y	Y	Y	Y	U
2002 Eleanor J. Barnes	Y	Y	Y	Y	Y	N	Y	Y
2025 Guiying Liu	Y	Y	Y	Y	Y	Y	Y	N
2025 Saravanan Krishinan	Y	Y	Y	Y	Y	Y	Y	Y
2024 Jin Y	Y	Y	Y	Y	Y	Y	Y	Y
2024 Aleksandra Klebukowska	Y	Y	Y	Y	Y	Y	Y	Y
2024 Federica Troisi	Y	Y	Y	Y	N	Y	Y	Y
2024 Nismat Javed	Y	Y	Y	Y	Y	Y	Y	Y
2024 Tao Li	Y	Y	Y	Y	Y	Y	Y	Y
2023 Changjin Li	Y	Y	Y	Y	Y	N	Y	Y
2023 Sara Mladoniczky	Y	Y	Y	Y	Y	Y	Y	Y
2023 Yang Bai	Y	Y	Y	Y	Y	Y	Y	Y
2023 Sharath C Vipparthy	Y	Y	Y	Y	Y	Y	Y	U

Y,yes; N,no; U,unclear.

Table S1. Characteristics of included studies reported in women with arrhythmia and electrical cardioversion was performed

Year	Author	Electrical shock energy	Gestation at cardioversion	Age	Pregnancy history	Type of arrhythmia	Symptom	Antiarrhythmic drug	Outcome
2023[1]	M. Cauldwell	NA	25.7w	30y	_	AF,AFL,AT,SVT	NA	NA	Success
2019[2]	N. Janjua	200J	35w	37y	G3P2	AF	Palpitations,breathless	Flecainide	Success
2016[3]	Matěcha, J	40J	37w	N/A	G2P1	AFL	None	None	Success
2016[4]	Rekha Agrawal	200J	37w	30y	G2P1	SVT	Palpitations	None	Success
2016[5]	Sungmin Lee	100-200J	20w	31y	G1P0	VT	Palpitations	None	Success
2014[6]	N. Murphy	NA	33w	37y	N/A	AF	Palpitations	Metoprolol	Success
2012[7]	Fevzi Yılmaz	100J	26w	32y	G2P1	SVT	Palpitations	Metoprolol,adenosine	Success
2011[8]	C. H. N. Tromp	50J	21w	34y	G3P2	AF	Palpitation, agitation	β-blocker	Success
2011[8]	C. H. N. Tromp	100-360J	34w	29y	G1P0	AT	Palpitations	Adenosine,verapamil	**Failure**
2011[9]	A. DiCarlo-Meacham	100J	22w	N/A	G1P0	AF	Chest pain	Metoprolol	Success
2011[10]	Charly J Sengheiser	100J	39w	26y	G2P1	AF	Chest tightness	Digoxin, bisoprolol	Success
2010[11]	John D	100-360J	27w	20y	G1P0	AT	Dyspnea	Beta-blockers	**Failure**
2009[12]	A Puri	100J,360J	36w	25y	G3P2	VT	Breathlessness	Adenosine, diltiazem	**Failure**
2007[13]	Chia-Hui Lin	20-100J	26w	35y	G3P1	SVT	Palpitations	Adenosine,verapamil	Success
2006[10]	Arimie R	NA	34w	24y	N/A	AF	NA	Diltiazem, digoxin	Success
2006[14]	Tsui-Hua Wu	NA	25w	30y	G1P0	AF	NA	Digoxin and sotalol	**Failure**
2006[15]	Parasuraman R	100J	33w	39y	N/A	AF	NA	None	Success
2002[16]	Eleanor J. Barnes	50J	28w	24y	G2P1	SVT	Palpitations	Adenosine,verapamil	Success
1983[17]	Cullhead I	80J-200J	21w	21y	G1P0	AF	Palpitation	Thiomebumal	Success
1981[18]	Klepper I	200J	35w	28y	G1P0	SVT	None	Verapamil,practolol	Success
1976[18]	Vinci and Mignone	250J	15w	N/A	N/A	AF	NA	None	Success
1973[19]	Grand and Bernard	300J	37w	N/A	N/A	AF	NA	None	Success
1973[19]	G.J.Robards	NA	23w	23y	G1P0	SVT	Fatigue,breathlessness	Digoxin,practolol	**Failure**
1971[19]	Johns. S	100J	8w	25y	G1P0	AT	Breathlessness	None	**Failure**
1969[19]	Palliez	150J	24w	N/A	N/A	AF	NA	None	Success
1969[19]	Palliez	200J	28w	N/A	N/A	AF	NA	None	Success
1969[19]	Palliez	100J,200J	12w	N/A	N/A	AF	NA	None	Success

Year	Author	Electrical shock energy	Gestation at cardioversion	Age	Pregnancy history	Type of arrhythmia	Symptom	Antiarrhythmic drug	Outcome
1969[19]	Palliez	250J	16w	N/A	N/A	AF	NA	None	Success
1966[19]	Sussmam	100J	7w	N/A	N/A	AFL	None	None	Success
1966[19]	Howard F	100J	7w	32y	G4P3	AFL	Weakness, dizziness	Digoxin,pronestyl	Success
1965[19]	Meitus	100J	7w	N/A	N/A	AFL	NA	None	Success
1965[19]	Vogel	100J	20w	N/A	N/A	AF	NA	None	Success

Abbreviation: SVT: Supraventricular tachycardia; AT:Atrial tachycardia; AF:Atrial fibrillation; AFL:Atrial flutter; VT: Ventricular tachycardia; VF: Ventricular fibrillation;

Table S2. Characteristics of included studies reported in women with arrhythmia and cather ablation was performed

Year	Author	Number		Age at conception	Pregnancy history	Gestation at ablation	Type of arrhythmia
2025[20]	Guiying Liu	1	CARTO	34	NA	32	VT
2025[21]	Saravanan Krishinan	6	CARTO	29.5	NA	19.6	PVC
2024[22]	Jin Y	1	CARTO	33	G1P0	20	SVT
2024[23]	Aleksandra Klebukowska	4	CARTO	27	NA	26	AVNRT,WPW
2024[24]	Federica Troisi	1	CARTO	34	NA	20	AT
2024[25]	Nismat Javed	1	NA	25	NA	NA	AT
2024[26]	Tao Li	1	NA	29	NA	21	PVC
2023[27]	Changjin Li	1	Ensite	25	G1P0	12	PVC
2023[28]	Sara Mladoniczky	13	CARTO, Ensite	30.3	NA	24	AT,AVRT,AVNRT,VT
2023[29]	Yang Bai	1	CARTO	26	NA	21	SVT
2023[30]	Sharath C Vipparthy	1	CARTO	36	G7P4	13	AF

Abbreviation: SVT: Supraventricular tachycardia; AF:Atrial fibrillation; AFL:Atrial flutter; VT: Ventricular tachycardia;AVNRT:atrioventricular nodal reentrant tachycardia; PVC:Premature ventricular contraction; WPW: Wolff Parkinson White; The remaining studies on catheter ablation during pregnancy, please refer to the research conducted by Shi-Min Yuan[31].

References：

[1] M. Cauldwell *et al.*, 'Direct current cardioversion in pregnancy: a multicentre study', *BJOG Int. J. Obstet. Gynaecol.*, vol. 130, no. 10, pp. 1269–1274, Sept. 2023, doi: 10.1111/1471-0528.17457.

[2] N. B. Janjua, S. A. Birmani, T. McDonagh, A. Hameed, and M. McKernan, 'New-onset lone maternal atrial fibrillation: a case report', *Medicine (Baltimore)*, vol. 99, no. 7, p. e19156, Feb. 2020, doi: 10.1097/MD.0000000000019156.

[3] J. Matěcha and L. Riedlbauchová, '[electrical cardioversion in pregnancy - case report]', *Ceska Gynekol.*, vol. 81, no. 1, pp. 38–40, Jan. 2016.

[4] R. Agrawal, H. Shintre, and B. Rani, 'A rare case of supraventricular tachycardia during pregnancy and successful management in crisis situation with electrical cardioversion and radiofrequency ablation', *J. Obstet. Gynaecol. India*, vol. 66, no. Suppl 2, pp. 594–597, Oct. 2016, doi: 10.1007/s13224-015-0836-0.

[5] S. Lee, 'Termination of idiopathic sustained monomorphic ventricular tachycardia by synchronized electrical cardioversion during pregnancy', *Acute Crit. Care*, vol. 33, no. 1, pp. 46–50, Feb. 2018, doi: 10.4266/acc.2016.00115.

[6] 'Case report on the treatment of atrial fibrillation in a pregnant woman of 33 weeks' gestation'. Accessed: Nov. 30, 2025. [Online]. Available: https://www.scirp.org/journal/paperinformation?paperid=41512

[7] F. Yılmaz, I. Beydilli, C. Kavalcı, and S. Yılmaz, 'Successful electrical cardioversion of supraventricular tachycardia in a pregnant patient', *Am. J. Case Rep.*, vol. 13, pp. 33–35, 2012, doi: 10.12659/AJCR.882594.

[8] C. H. N. Tromp, A. C. M. Nanne, P. J. M. Pernet, R. Tukkie, and A. C. Bolte, 'Electrical cardioversion during pregnancy: safe or not?', *Neth. Heart J. Mon. J. Neth. Soc. Cardiol. Neth. Heart Found.*, vol. 19, no. 3, pp. 134–136, Mar. 2011, doi: 10.1007/s12471-011-0077-5.

[9] L. A. DiCarlo-Meacham and L. J. Dahlke, 'Atrial fibrillation in pregnancy', *Obstet. Gynecol.*, vol. 117, no. 2 Pt 2, pp. 489–492, Feb. 2011, doi: 10.1097/AOG.0b013e31820561ef.

[10] C. J. Sengheiser and K. C. Channer, 'Recurrent atrial flutter and fibrillation in pregnancy', *BMJ Case Rep.*, vol. 2011, p. bcr1220103589, June 2011, doi: 10.1136/bcr.12.2010.3589.

[11] J. D. Ferguson, A. Helms, J. M. Mangrum, and J. P. DiMarco, ‘Ablation of incessant left atrial tachycardia without fluoroscopy in a pregnant woman’, *J. Cardiovasc. Electrophysiol.*, vol. 22, no. 3, pp. 346–349, Mar. 2011, doi: 10.1111/j.1540-8167.2010.01847.x.

[12] A. Puri *et al.*, ‘Peripartum cardiomyopathy presenting with ventricular tachycardia: a rare presentation’, *Indian Pacing Electrophysiol. J.*, vol. 9, no. 3, pp. 186–189, May 2009.

[13] C.-H. Lin and C.-N. Lee, ‘Atrial fibrillation with rapid ventricular response in pregnancy’, *Taiwan. J. Obstet. Gynecol.*, vol. 47, no. 3, pp. 327–329, Sept. 2008, doi: 10.1016/S1028-4559(08)60133-3.

[14] T.-H. Wu, L.-C. Huang, M. Ho, C.-C. Lee, T.-H. Chiu, and Y.-C. Hung, ‘Fetal atrial flutter: a case report and experience of sotalol treatment’, *Taiwan. J. Obstet. Gynecol.*, vol. 45, no. 1, pp. 79–82, Mar. 2006, doi: 10.1016/S1028-4559(09)60199-6.

[15] R. Parasuraman, M. M. Gandhi, and N. H. Liversedge, ‘Nifedipine tocolysis associated atrial fibrillation responds to DC cardioversion’, *BJOG Int. J. Obstet. Gynaecol.*, vol. 113, no. 7, pp. 844–845, July 2006, doi: 10.1111/j.1471-0528.2006.00964.x.

[16] E. J. Barnes, F. Eben, and D. Patterson, ‘Direct current cardioversion during pregnancy should be performed with facilities available for fetal monitoring and emergency caesarean section’, *BJOG Int. J. Obstet. Gynaecol.*, vol. 109, no. 12, pp. 1406–1407, Dec. 2002, doi: 10.1046/j.1471-0528.2002.02113.x.

[17] I. Cullhed, ‘Cardioversion during pregnancy. A case report’, *Acta Med. Scand.*, vol. 214, no. 2, pp. 169–172, 1983.

[18] I. Klepper, ‘Cardioversion in late pregnancy. The anaesthetic management of a case of wolff-parkinson-white syndrome’, *Anaesthesia*, vol. 36, no. 6, pp. 611–616, June 1981, doi: 10.1111/j.1365-2044.1981.tb10325.x.

[19] K. Ueland, J. H. McAnulty, F. R. Ueland, and J. Metcalfe, ‘Special considerations in the use of cardiovascular drugs’, *Clin. Obstet. Gynecol.*, vol. 24, no. 3, pp. 809–823, Sept. 1981, doi: 10.1097/00003081-198109000-00008.

[20] G. Liu, S. Quan, X. Chen, and X. Zhang, ‘Emergency zero-fluoroscopy catheter ablation for refractory ventricular tachycardia in third-trimester pregnancy: a case report’, *Front. Cardiovasc. Med.*, vol. 12, p. 1668549, 2025, doi: 10.3389/fcvm.2025.1668549.

[21] S. Krishinan *et al.*, ‘Maternal and fetal outcomes in zero fluoroscopy symptomatic PVC ablation’, *JACC Case Rep.*, vol. 30, no. 28, p. 105073, Sept. 2025, doi: 10.1016/j.jaccas.2025.105073.

[22] Y. Jia, H. Liao, Q. Hu, H. Liu, Z. Zeng, and H. Yu, ‘Catheter ablation in a monochorionic diamniotic twin pregnancy: a case report and literature review’, *Medicine (Baltimore)*, vol. 103, no. 44, p. e40443, Nov. 2024, doi: 10.1097/MD.0000000000040443.

[23] A. Kłębukowska, P. Futyma, Ł. Zarębski, J. Sander, M. Futyma, and P. Kułakowski, ‘Nonfluoroscopic catheter ablation of supraventricular tachycardias during pregnancy using simplified electroanatomic marker annotation’, *Hear. Case Rep.*, vol. 10, no. 12, pp. 953–959, Dec. 2024, doi: 10.1016/j.hrcr.2024.09.011.

[24] F. Troisi *et al.*, ‘Zero-fluoroscopy catheter ablation of right appendage focal atrial tachycardia in a pregnant woman’, *Clin. Pract.*, vol. 14, no. 3, pp. 946–953,

May 2024, doi: 10.3390/clinpract14030075.

[25] N. Javed, S. Ashraf, A. Gore, M. Aziz, M. A. Aziz, and E. Sklyar, ‘A case of atrial tachycardia masquerading as sinus tachycardia in a pregnant female-a case report’, *Case Rep. Cardiol.*, vol. 2024, p. 5523100, 2024, doi: 10.1155/cric/5523100.

[26] T. Li, X. Li, L. Zhou, M. A. U. Hassan, and Z. Yang, ‘Ventricular premature beats in a pregnant woman originating from trabeculae carneae in the right ventricle treated with radiofrequency ablation without X-ray guidance: a case report’, *J. Med. Case Reports*, vol. 18, no. 1, p. 587, Nov. 2024, doi: 10.1186/s13256-024-04951-z.

[27] C. Li *et al.*, ‘Zero x-rays radiofrequency catheter ablation for ventricular premature contraction originating from the left coronary cusp during pregnancy: a case report’, *Front. Cardiovasc. Med.*, vol. 10, Sept. 2023, doi: 10.3389/fcvm.2023.1183787.

[28] S. Mladoniczky *et al.*, ‘Case series of catheter-based arrhythmia ablation in 13 pregnant women’, *Clin. Cardiol.*, vol. 46, no. 8, pp. 942–949, Aug. 2023, doi: 10.1002/clc.24072.

[29] Y. Bai, J. Qiu, M. Hu, and G. Chen, ‘Emergent zero-fluoroscopy mapping and thoracoscopic ectomy of appendage in pregnant women with life-threatening atrial tachycardia: a case report and literature review’, *Med. Kaunas Lith.*, vol. 59, no. 3, p. 528, Mar. 2023, doi: 10.3390/medicina59030528.

[30] S. C. Vipparthy, J. Gomez, and A. K. Mehrotra, ‘Zero-fluoroscopy pulmonary vein isolation with intracardiac echocardiography to monitor fetus in pregnant patient with atrial fibrillation’, *Hear. Case Rep.*, vol. 9, no. 2, pp. 76–79, Feb. 2023, doi: 10.1016/j.hrcr.2022.10.020.

[31] S.-M. Yuan, ‘Catheter ablation for tachyarrhythmias during pregnancy’, *Postepy W Kardiologii Interwencyjnej Adv. Interv. Cardiol.*, vol. 18, no. 3, pp. 206–216, Sept. 2022, doi: 10.5114/aic.2022.122032.
